# Sound Absorption Characteristics of Aluminum Foams Treated by Plasma Electrolytic Oxidation

**DOI:** 10.3390/ma8115395

**Published:** 2015-11-09

**Authors:** Wei Jin, Jiaan Liu, Zhili Wang, Yonghua Wang, Zheng Cao, Yaohui Liu, Xianyong Zhu

**Affiliations:** 1 Key Laboratory of Automobile Materials (Ministry of Education), College of Materials Science and Engineering, Jilin University, Changchun 130022, China; Jinweiking2005@163.com (W.J.); Wangzl@mail.jlu.edu.cn (Z.W.); Caozheng@163.com (Z.C.); Liuyh@mail.jlu.edu.cn (Y.L.); Zhuxy@jlu.edu.cn (X.Z.); 2 Key Laboratory of Bionic Engineering (Ministry of Education), Jilin University, Changchun 130022, China; Wangyh@mail.jlu.edu.cn; 3 School of Mechanical and Electric Engineering, Changchun University of Science and Technology, Changchun 130022, China

**Keywords:** foams, aluminum, plasma electrolytic oxidation, sound absorption

## Abstract

Open-celled aluminum foams with different pore sizes were fabricated. A plasma electrolytic oxidation (PEO) treatment was applied on the aluminum foams to create a layer of ceramic coating. The sound absorption coefficients of the foams were measured by an impedance tube and they were calculated by a transfer function method. The experimental results show that the sound absorption coefficient of the foam increases gradually with the decrease of pore size. Additionally, when the porosity of the foam increases, the sound absorption coefficient also increases. The PEO coating surface is rough and porous, which is beneficial for improvement in sound absorption. After PEO treatment, the maximum sound absorption of the foam is improved to some extent.

## 1. Introduction

Aluminum foams with open-cell structure have many outstanding physical and mechanical properties, such as low density, high damping properties, and excellent sound absorption [1,2]. These advantages make aluminum foams suitable for application in many fields, including transportation, electronics, and the aerospace industry [1,2,3].

There are increasing studies on the sound absorption characteristics of the aluminum foams [4,5,6,7,8,9]. Lu *et al.* presented an experimental and theoretical study for the sound absorption of aluminum foams with semi-open cells. The theoretical model revealed the correlation between sound absorption and morphological parameters such as pore size, pore opening, and porosity [4]. Li *et al.* fabricated aluminum foams with spherical cells by an infiltration process. They found sound absorption increases with an increase in the number of pore openings or with a decreased diameter of the pore openings [5]. Wang found that the sound absorption behavior of open-celled aluminum foam was correlated with the pore size, sample thickness, and backing air gap depth. As frequency or sample thickness increases, the sound absorption is significantly enhanced [6]. Masataka Hakamada fabricated the aluminum foams with porosities of 85%–95% by the spacer method. The sound absorption coefficient increased with porosity and thickness. However, they found that there was no apparent correlation between the pore size and sound absorption coefficient [7]. The sound absorption coefficient of the aluminum is significantly improved by inserting an air gap between the tested sample and the back surface [5,8]. Recently, Ren synthesized ZnO micro-rods on the cell walls of open-celled aluminum foams. The experiment showed that the sound absorption coefficient increased about 40% at maximum, which indicates that the sound absorption characteristics of aluminum foams can be improved by surface treatment [9].

Though some studies were carried out on the sound absorption of aluminum foams, the studies for the purpose of improving the sound absorption by using a surface treatment method are still shown to be deficient. Therefore, in this study, the plasma electrolytic oxidation (PEO) treatment [10], also named micro-arc oxidation (MAO) [11,12], was applied on the aluminum foams, and the morphology, structure, and sound absorption characteristic of the foams were investigated.

## 2. Results and Discussion

### 2.1. Morphology of the Foams

**Figure 1:**
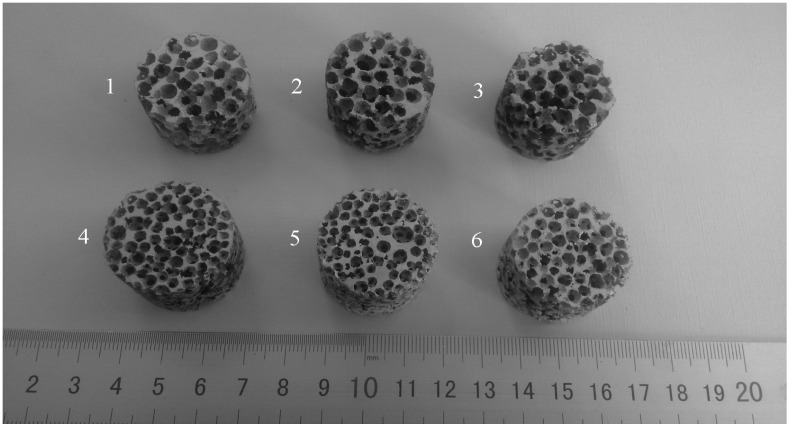
Typical morphology of the Al foams used for the sound absorption test.

**Figure 2:**
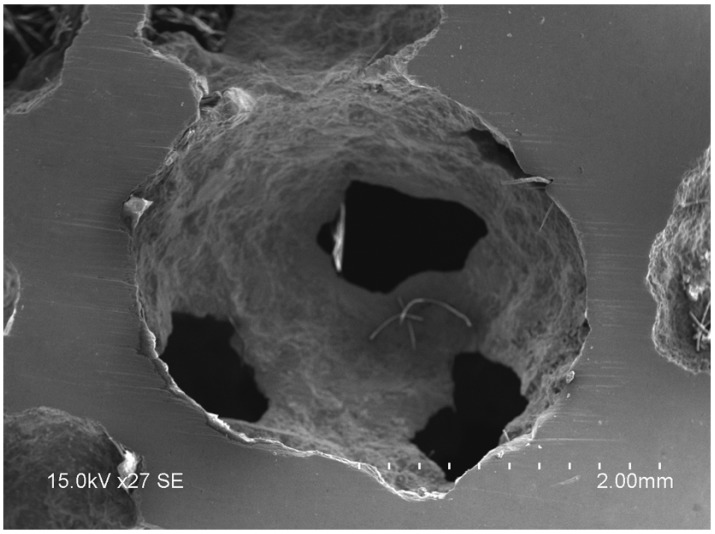
SEM of typical microstructure of the Al foams.

Figure 1 shows the typical morphology of the Al foams. The pores of the foams are spheroidal. These foams have different pore sizes which were counted by digital image software. The foams are classified by the mean pore size for the measurements of the sound absorption coefficient. The mean pore sizes of the foams marked digitally from 1 to 6 are ~4.48, ~5.18, ~5.70, ~3.52, ~3.08, and ~3.32 mm, respectively. Additionally, the porosities of the foams marked digitally from 1 to 6 are ~65%, ~76%, ~77%, ~75%, ~73%, and ~66%, respectively. Figure 2 shows the microstructure of the Al foams. It can be seen from Figure 2 that the small pore openings exist on the cell walls that form the interconnected channels among the foams' pores, which is similar to the foams reported by Li [5].

### 2.2. Microstructure of the PEO Coating

**Figure 3:**
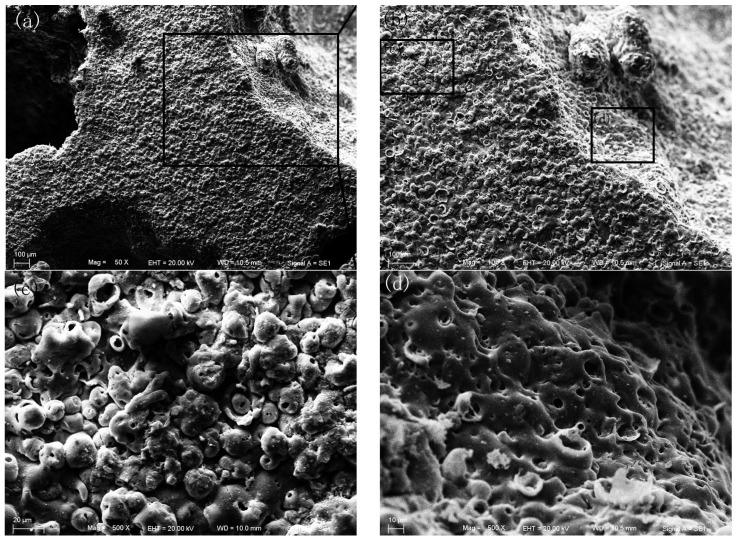
SEM images of the PEO coatings on the foams: (**b**) magnification of (**a**), (**c**) and (**d**) magnification of (**b**).

**Figure 4:**
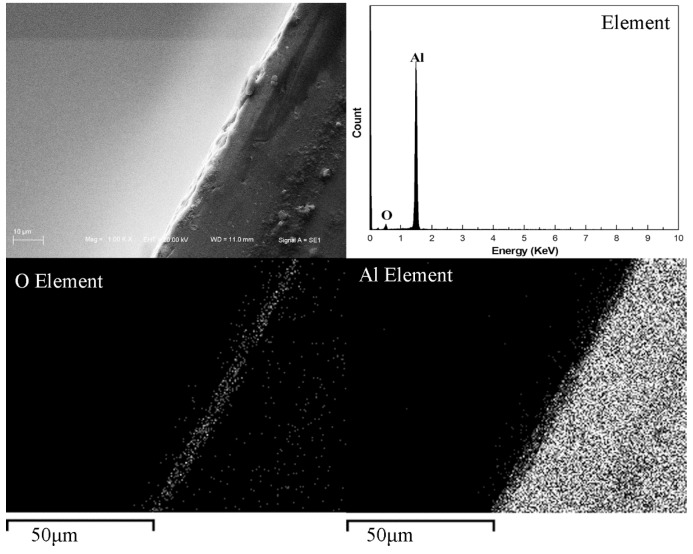
SEM image of cross-section of PEO coating and its EDS analysis.

The SEM images of PEO coatings on the foams are shown in Figure 3. It can be seen that the coating surface is rough and porous, *i.e.*, there are some micro-pores on the surface, which results from the micro-arc discharges [13,14,15,16]. During the PEO process, the micro-arc discharges happen constantly. When a micro-arc extinguishes, it will leave a micro-pore on the surface. Therefore, the PEO coatings show a porous and rough surface. Figure 4 shows an SEM image of a cross-section of PEO coating and its EDS analysis. It can be seen that the PEO coatings are mainly composed of aluminum and oxygen. Additionally, the thickness of the PEO coating is about 6.3 μm. Therefore, after PEO treatment, the pore size change can be negligible in the present Al foams.

### 2.3. XRD Study of the PEO Coating

**Figure 5:**
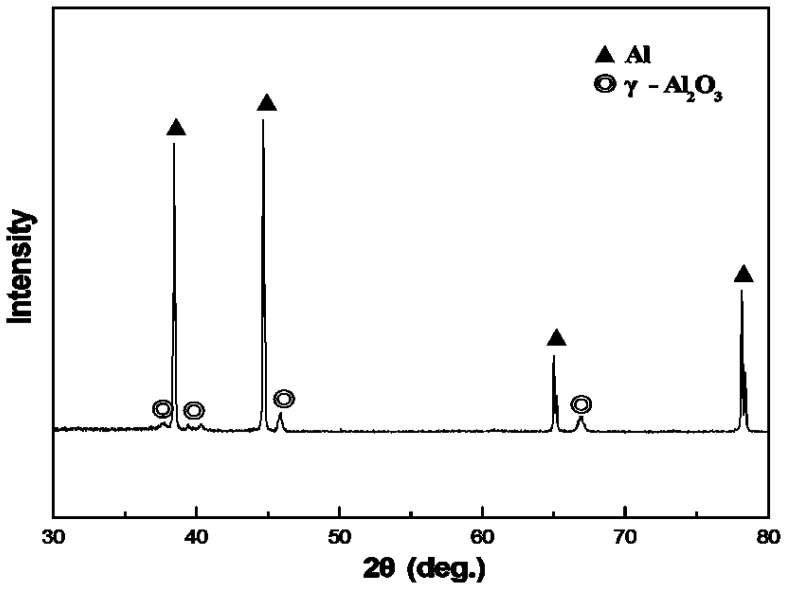
XRD pattern of the PEO-coated Al foams.

The XRD diffraction pattern of the coated Al foams is shown in Figure 5. It is clear that the major phase in the PEO coatings is γ-Al_2_O_3_. The presence of the Al pattern is due to the substrate material.

### 2.4. Sound Absorption Characteristics

#### 2.4.1. Effect of Pore Size on the Sound Absorption of the Foams

**Figure 6:**
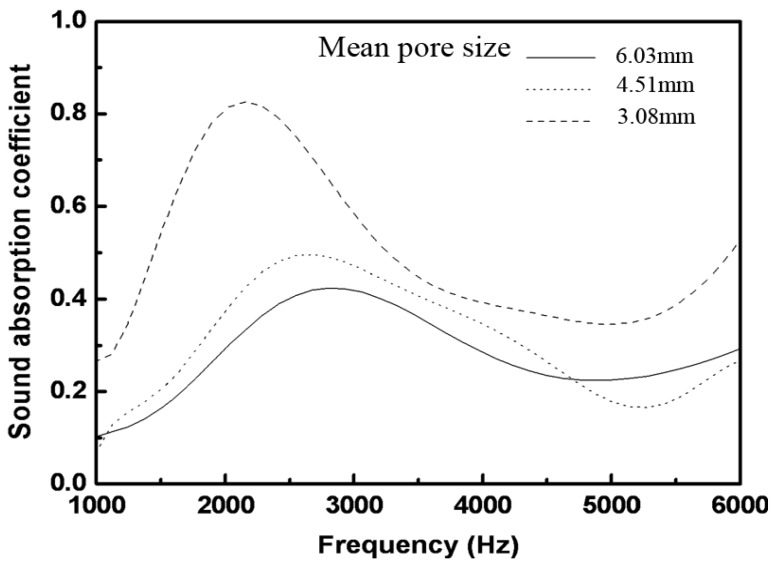
Sound absorption coefficient *vs.* frequency of the foams with different mean pore sizes.

Figure 6 shows the sound absorption of the foams with different pore sizes and approximately uniform porosities (around 73%) and thicknesses (around 2.25 cm) in the frequency range of 1000–6000 Hz. From Figure 6, it can be concluded that the sound absorption of the foams is increasing with decreasing pore sizes. Therefore, it is concluded that relatively small pores of the foams are beneficial to sound absorption. This tendency is consistent with those observed in aluminum foams [4,5,6].

#### 2.4.2. Effect of Porosity on Sound Absorption of the Foams

**Figure 7:**
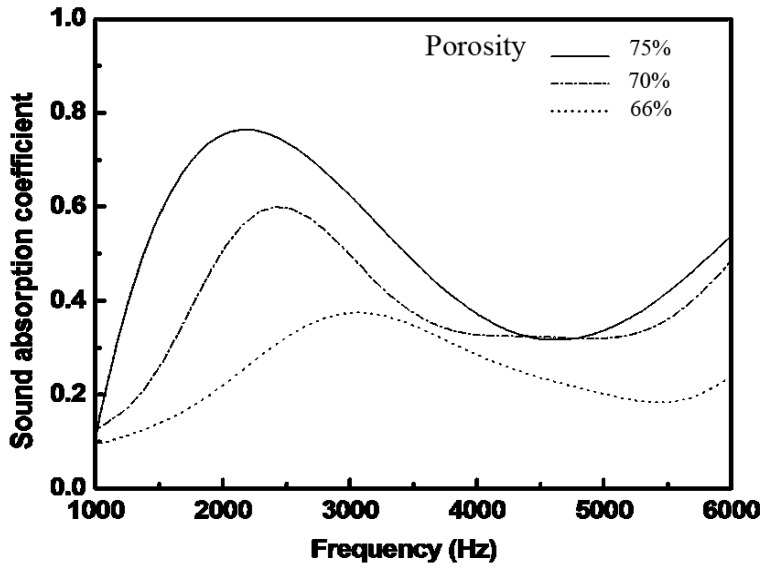
Sound absorption coefficient *vs.* frequency of the foams with different porosities.

Figure 7 shows the sound absorption curves of the foams with different porosities and approximately uniform mean pore sizes (around 5.7 mm) and thicknesses (around 2.7 cm). As shown in Figure 7, the sound absorption coefficient increases when the porosity changes from 66% to 75%, demonstrating that the foam with high porosity is a good sound absorber.

#### 2.4.3. Effect of PEO Treatment on Sound Absorption of the Foams

**Figure 8:**
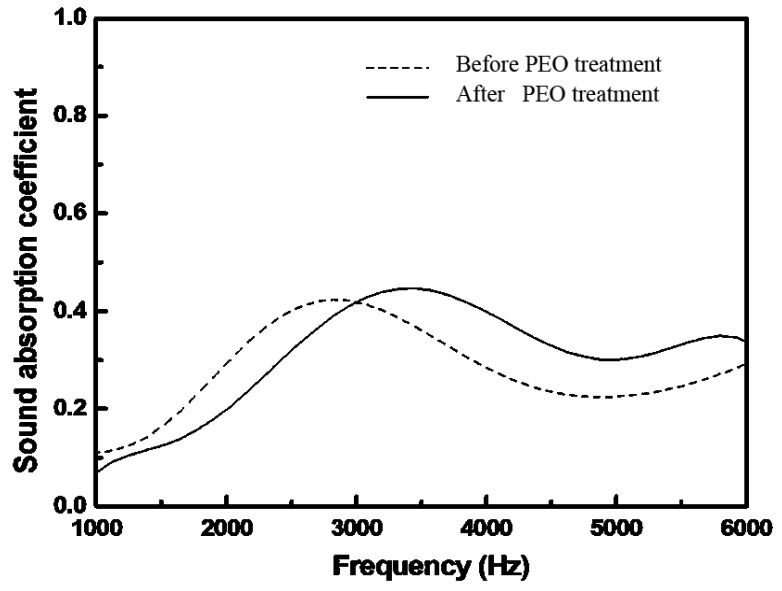
Sound absorption coefficient *vs.* frequency of the foams with PEO treatment.

Figure 8 shows the sound absorption curves of the foams with PEO treatment. It can be found that the sound absorption peak increased by ~5.5% after the PEO treatment. As mentioned above, two mechanisms are responsible for the sound absorption characteristics of Al foams, *i.e.*, the viscous losses resulting from the air flow and the thermal losses originating from the friction between the air and the cell wall through the vibration of air in the pores [4,5,6,9]. It can be found after the PEO treatment that there are small pores at micrometer level distributed in the surface of the aluminum foams. These small pores add a transport path of the sound wave and increase the flow resistance of the sound wave. Therefore, it is obvious that the rough and porous coating will enhance the interaction between the air and the pore, not only in the friction force but also the thermal exchange, which raises the sound wave loss. It is also noted that the displacement of the absorption peak shifted to the right a bit. It can be inferred that the displacement of the absorption peak might be attributed to the complex effect of change of the foam structure.

## 3. Experimental Section

### 3.1. Preparation and PEO Treatment of the Foams

Pure aluminum was used as raw material to prepare the foams. The calcium chloride preform was fabricated according to the following procedure: the spheroidal calcium chloride particles (>99% pure) were blended with inorganic binder in a mold to form a mixture, which was later dried out at about 260–350 °C. The porous preform was then sintered at about 580–650 °C. To make the foams, the calcium chloride preform was infiltrated with the molten Al melted at about 680–740 °C. Different process parameters could be applied to obtain Al foams with different densities and pore sizes [17].

The foam samples were PEO-coated using an alternating current power source and an alkali electrolyte. The electrolysis environment was an aqueous electrolyte containing NaOH and Na_2_SiO_3_ at concentrations of 4 g/L and 9 g/L, respectively. The working frequency of PEO oxidation experiments was 500 Hz. During the coating process, the temperature of the electrolyte was maintained constant at approximately 30 °C using a stirring and cooling system [18].

### 3.2. Material Characterization

The porosities of the Al foams were calculated using the following equation:

(1) *P* = (1−ρ^*^/ρ_s_) × 100%

where *P* is the porosity of the foams; ρ^*^ and ρ_s_ are the densities of the foams and the cell wall material, respectively; and ρ^*^/ρ_s_, which is called the relative density of the foams, indicates the ratio of the density of the foams to the density of the cell wall material.

The pore size of the foam was obtained by digital image processing software (Nano Measurer series) from optical images of the foams. The microstructure of the foams and PEO coating were observed by scanning electron microscopy (SEM) (Model JSM-5310, Tokyo, Japan). The different elements in the composites were analyzed by the energy-dispersive X-ray spectroscopy (EDS) (Model X-Max, Oxford, UK). The phases were analyzed by X-ray diffraction (XRD) (Model D/Max 2500PC Rigaku, Tokyo, Japan). The wavelength used was Cu Ka and it was scanned from 30° to 80° with a scan speed of 1 s/step.

### 3.3. Measurements of Sound Absorption Coefficient

**Figure 9:**
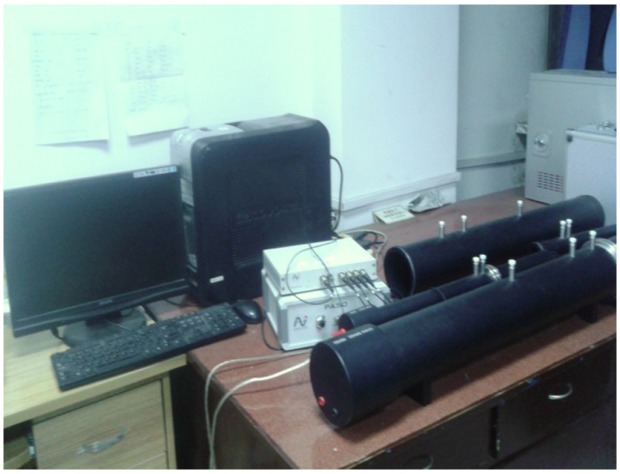
The impedance tube devices.

There are two main types of methods for determining the absorption coefficient of acoustic materials: the reverberation time method for a diffuse field, and the impedance tube method for a normal incidence. In this investigation, the sound absorption coefficients of foams were measured by using the impedance tube. Therefore, the “absorption coefficient” measured in this study is the “sound absorption coefficient at normal incidence”. The impedance tube is manufactured by BSWA TECH (SW series), as shown in Figure 9. Broadband random sound waves were generated by the loudspeaker at one end of the impedance tube and transmitted to the surface of the sample at the other end. The reflected signals were picked up by the sensors. The diameter of the tested sample is 30 mm. The date was measured by applying the transfer function method due to its reproducibility. The data collected by the computer system were then used to plot the sound absorption coefficient frequency (1000–6000 Hz) curves. In order to ascertain reproducibility, each tested result was the average obtained from more than three tested specimens.

## 4. Conclusions

In this paper, the sound absorption characteristics of aluminum foams have been evaluated. PEO treatment was applied on the foams to deposit a layer of coating. The PEO coating is rough and porous. In the PEO coating, γ-Al_2_O_3_ is present as the major phase. The sound absorption increases gradually with the decrease of pore size or the rise of porosity. The maximum sound absorption coefficient increases by ~5.5% after the PEO treatment. The improvement is attributed to the micro-pores and the rough surface that enhances the sound wave loss by increasing the friction between the surface and the air.
